# Brain-Derived Neurotrophic Factor Is Involved in Activity-Dependent Tonotopic Refinement of MNTB Neurons

**DOI:** 10.3389/fncir.2022.784396

**Published:** 2022-02-03

**Authors:** Mackenna Wollet, Jun Hee Kim

**Affiliations:** Department of Cellular and Integrative Physiology, UT Health San Antonio, San Antonio, TX, United States

**Keywords:** BDNF, auditory brainstem, MNTB, tonotopy, axon initial segment

## Abstract

In the mammalian brain, auditory brainstem nuclei are arranged topographically according to acoustic frequency responsiveness. During postnatal development, the axon initial segment (AIS) of principal neurons undergoes structural refinement depending on location along the tonotopic axis within the medial nucleus of the trapezoid body (MNTB). However, the molecular mechanisms underlying the structural refinement of the AIS along the tonotopic axis in the auditory brainstem have not been explored. We tested the hypothesis that brain-derived neurotrophic factor (BDNF) is a molecular mediator of the structural development of the MNTB in an activity-dependent manner. Using *BDNF* heterozygous mutant (*BDNF^+/–^*) mice, we examined the impact of global BDNF reduction on structural and functional development of MNTB neurons by assessing AIS structure and associated intrinsic neuronal properties. BDNF reduction inhibits the structural and functional differentiation of principal neurons along the tonotopic axis in the MNTB. Augmented sound input during the critical period of development has been shown to enhance the structural refinement of the AIS of MNTB neurons. However, in *BDNF^ +/–^* mice, MNTB neurons did not show this activity-dependent structural modification of the AIS following repeated sound stimulation. In addition, *BDNF^+/–^* mice lacked a defined isofrequency band of neuronal activity following exposure to 16 kHz sound, suggesting degradation of tonotopy. Taken together, structural development and functional refinement of auditory brainstem neurons require physiological levels of BDNF to establish proper tonotopic gradients.

## Introduction

Along the auditory processing pathway, the topographic organization of neurons is important for determining where sound frequencies are processed within each auditory nucleus. In the MNTB, one of the key sound localization nuclei in the auditory brainstem, neurons are arranged with graded frequency-responsiveness (from high- to low-frequency) along the medio-lateral axis, respectively. Physiological factors including ion channel expression and cell morphology (e.g., soma size) are graded along the tonotopic axis in brainstem nuclei (Weatherstone et al., 2017; Akter et al., 2018). For example, voltage-gated potassium channel (K_V_3) is highly expressed in high-frequency responding neurons, and expression decreases moving toward low-frequency responding neurons in a graded fashion in the mouse and avian brainstem (Li et al., 2001; Parameshwaran et al., 2001; von Hehn et al., 2004; Leao et al., 2006). In contrast to K_V_3 channels, K_V_1 channels have the opposite expression pattern within the MNTB with the highest K_V_1 density laterally (Leao et al., 2006). In terms of cell morphology, the soma size of MNTB neurons is different along the tonotopic axis, where lateral neurons are larger than medial neurons (Weatherstone et al., 2017). In addition, the length and location of the AIS, a key axonal domain responsible for action potential (AP) initiation and neuronal excitability, is also dependent on cell location along the tonotopic axis in the chick and mouse (Kuba et al., 2006, 2014; Kim et al., 2019). Tonotopic refinement of the AIS is impaired in deaf animals- either congenitally or *via* cochlear removal (Kuba et al., 2010; Kim et al., 2019). Oppositely, AIS tonotopic differentiation is enhanced following increased neural activity driven by acoustic enrichment during the critical development period in mice (Kim et al., 2019). The structural refinement of the AIS is dependent on tonotopic location and requires sound-evoked activity in the auditory brainstem. The AIS determines neuronal excitability and modulates neuronal output, thus can control auditory processing along the ascending auditory pathway (Kuba et al., 2006; Grubb and Burrone, 2010). However, the molecular mechanisms driving establishment of the tonotopic gradient of the AIS in the MNTB are unknown.

Previous work in cultured hippocampal neurons showed BDNF signaling regulates AIS location and affects neuronal excitability (Guo et al., 2017). Here, we investigated whether BDNF, an essential molecule for activity-dependent plasticity, is a molecular mediator for establishing tonotopic gradients of the AIS and associated neuronal properties in the auditory brainstem. BDNF expression begins in the inner ear at postnatal day 4 (P4) and the expression pattern follows the ascending pathway during development (Hafidi, 1999; Wiechers et al., 1999). By P14, *BDNF* mRNA expression is arranged tonotopically within the cochlea where expression is highest in the apical and medial turns, opposite of the *NT3* mRNA expression gradient (Schimmang et al., 2003). Acoustic enrichment increases BDNF transcript levels and protein levels in rodent brainstem due to increased neuronal activity (Wang et al., 2011; Matt et al., 2018). Using anti-BDNF antibodies to neutralize BDNF signaling, activity-dependent plasticity of tonotopy following pure tone sound stimulation was prevented in the rat auditory cortex (Anomal et al., 2013). We studied the effects of globally reduced BDNF levels on structural and intrinsic properties of MNTB neurons along the tonotopic axis in *BDNF^+/–^* mice. Given the role of BDNF in sound-evoked activity, tonotopic plasticity, and structural differentiation, we found that BDNF is one of the molecular mediators responsible for establishing structural tonotopic gradients and related physiological properties of auditory brainstem neurons during postnatal development.

## Materials and Methods

### Animals

**Figure 1:**
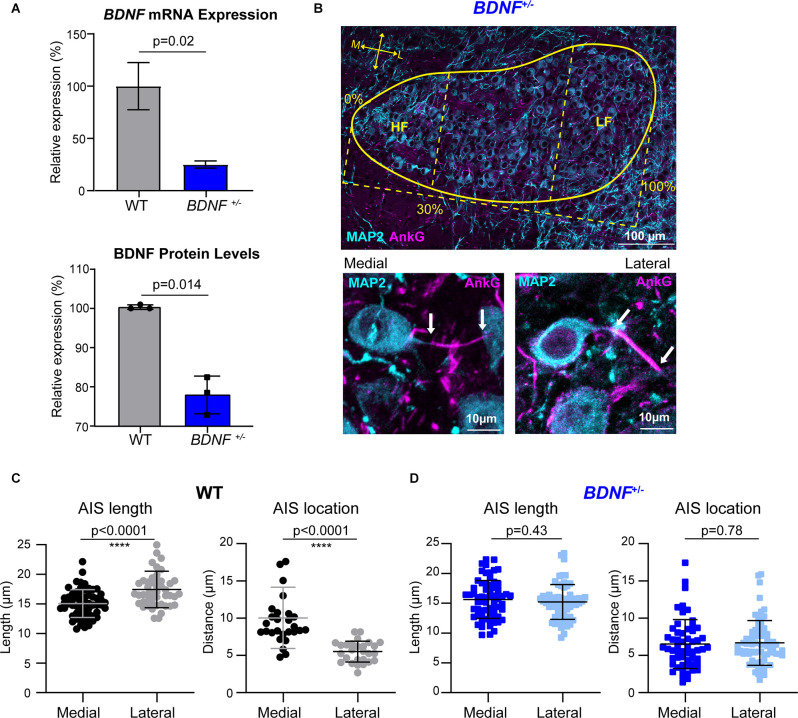
The tonotopic arrangement of the AIS in MNTB neurons is abolished in *BDNF^+/–^* mice. **(A)** *BDNF* mRNA expression (top) and BDNF protein level (bottom) in the MNTB from WT and *BDNF^+/–^* mice using qPCR and Western Blot, respectively. *BDNF* mRNA expression and protein level from *BDNF^+/–^* mice were normalized by those from WT (indicated by Relative expression, %). **(B)** The MNTB (at P20, the yellow line indicates border) was immunostained with MAP2 (cyan) and AnkG (magenta). HF indicates the high-frequency responding region, 30% of the medial (M) MNTB, and LF indicates the low-frequency responding region, 30% of the lateral (L) MNTB. (Bottom) Magnified images of medial and lateral MNTB neurons of *BDNF^+/–^* mice. Arrows indicate the proximal and distal ends of AIS. **(C)** Summary of length (μm) and location (distance from the soma) of the AIS in WT MNTB neurons. **(D)** AIS length and distance from soma from *BDNF^+/–^* medial and lateral neurons. Each point represents individual cells from three WT mice and four *BDNF^+/–^* mice. All error bars represent mean ± SD. **** indicates *p* < 0.0001.

**Figure 2:**
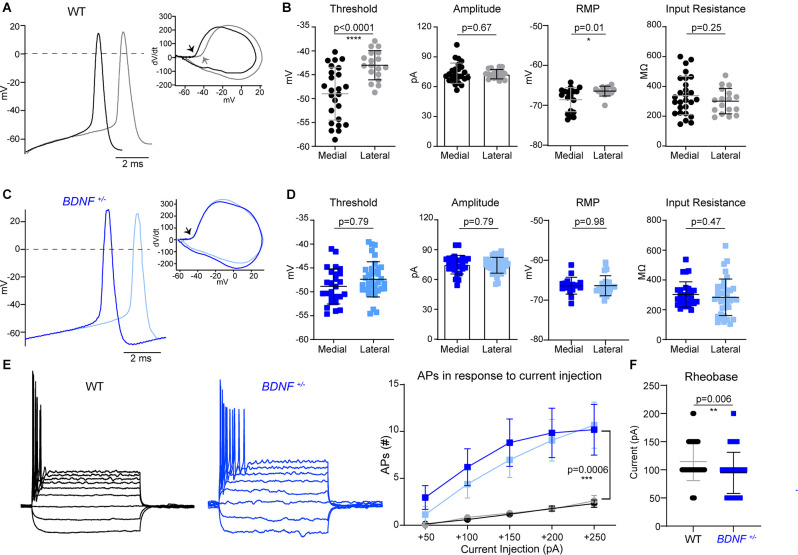
The difference in MNTB neuron intrinsic properties along the tonotopic axis is abolished in *BDNF^+/–^* mice. **(A)** Representative traces of APs evoked by current injection from medial (black) and lateral (gray) MNTB neurons in WT. Inset, dV/dt phase plot against membrane potential. The arrow points to the AP threshold, which differs between medial and lateral WT neurons. **(B)** Summary of AP threshold; determined as the membrane potential at dV/dt = 10, AP amplitude; determined as the difference between threshold and peak potential of AP, resting membrane potential, and input resistance of WT neurons. Each point represents an individual cell from 13 mice (medial) and seven mice (lateral). * indicates *p* < 0.05 and **** indicates *p* < 0.0001. **(C)** Representative traces of APs from *BDNF^+/–^* medial (dark blue) and lateral (light blue) neurons. dV/dt phase plot shows no difference in AP threshold between medial and lateral. **(D)** Summary of AP threshold, amplitude, resting membrane potential, and input resistance from *BDNF^+/–^* MNTB neurons. *n* = 10 mice (medial) and nine mice (lateral). **(E)** Membrane potential changes in WT and *BDNF^+/–^* neurons in response to step-like current injections from −100 pA to +250 pA in increments of 50 pA (left). The number of spikes in response to depolarizing current injection (right). Repeated measures ANOVA displayed significant difference based on genotype (*F*_(1, 99)_ = 12.55; *p* = 0.0006). *** indicates *p* < 0.001. **(F)** Rheobase current was quantified by genotype in WT and *BDNF^+/–^* neurons. ** indicates *p* < 0.01.

**Figure 3:**
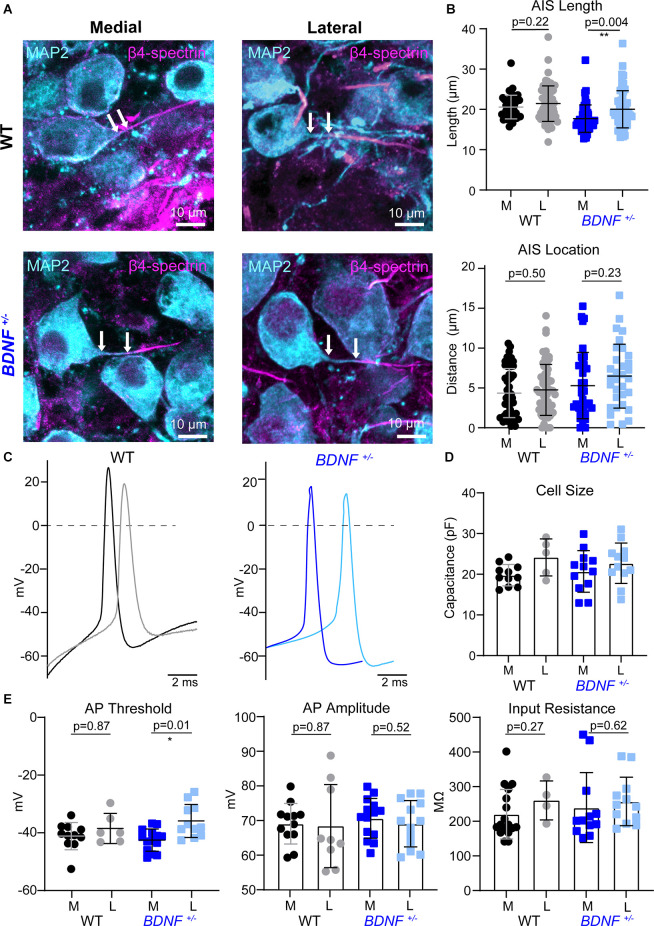
AIS structures and intrinsic properties of MNTB neurons at pre-hearing age. **(A)** MNTB neurons (MAP2, cyan) and corresponding AIS (β4-spectrin, magenta) of WT and *BDNF^+/–^* mice (at P9). Scale bar represents 10 μm. Arrows point to the edge of the soma and proximal end of the AIS, indicating the distance of the AIS. **(B)** Quantification of AIS location and length according to genotype and tonotopic location. Each point represents an individual cell from three mice/group. ** indicates *p* < 0.01. **(C)** Representative AP traces of medial and lateral neurons from WT and *BDNF^+/–^*mice. **(D)** Individual cell size was quantified by capacitance (pF) measurements during whole-cell recordings. Each point represents an individual cell. **(E)** Summary of AP threshold, amplitude, and input resistance by genotype and tonotopic location from five WT mice and four *BDNF^+/–^* mice. * indicates *p* < 0.05.

**Figure 4:**
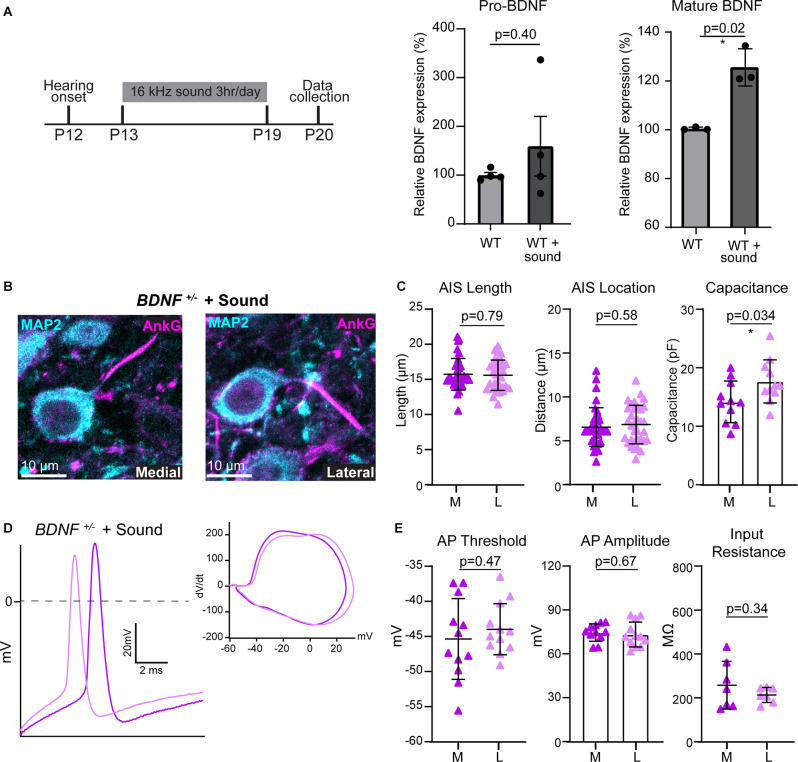
BDNF is necessary for the activity-dependent plasticity of MNTB neurons. **(A)** Sound stimulation paradigm schematic; 16 kHz sound for 3 h/day from P13 to P19 followed by data collection and mature and pro-BDNF protein levels in WT and WT + sound. * indicates *p* < 0.05. **(B)** MNTB neurons of *BDNF^+/–^* mice following sound stimulation were immunostained with anti-MAP2 (cyan) and anti-AnkG (magenta). **(C)** AIS length and location, and cell size were quantified by condition and tonotopic location. Each point represents individual cells from four mice for *BDNF^+/–^* and three mice for *BDNF^+/–^* + sound. **(D)** Representative AP traces from *BDNF^+/–^* medial (dark purple) and lateral (light purple) neurons after sound stimulation. Inset, dV/dt phase plot against membrane potential. **(E)** Summary of AP threshold, amplitude, and input resistance were quantified for each condition and tonotopic location. Each point represents an individual cell.

**Figure 5:**
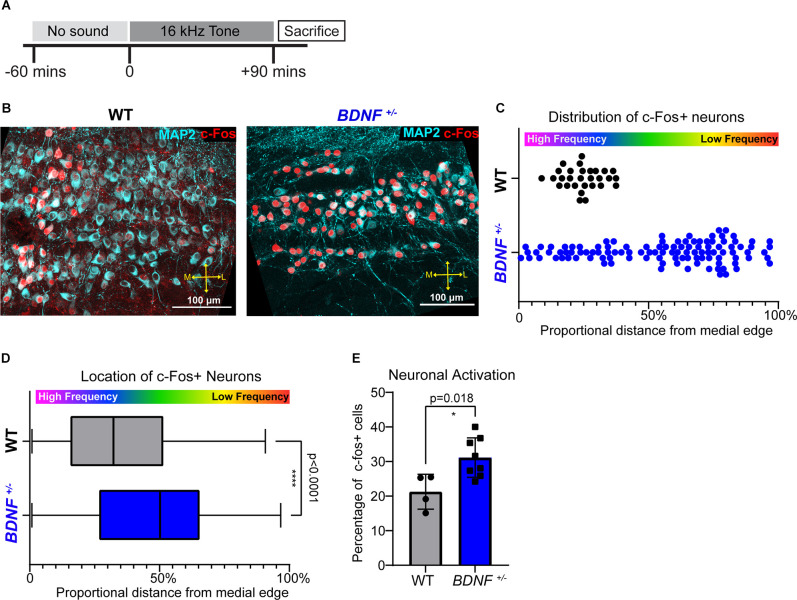
High frequency sound responding neurons is not tonotopically organized in *BDNF^+/–^* mice. **(A)** Tone exposure paradigm schematic. P21 mice were exposed to a pure 16 kHz tone for 90 min preceded by 60 min of silence in a sound-attenuated chamber. **(B)** Representative image of the MNTB immunostained with MAP2 (cyan) and c-Fos (red). Scale bars represent 100 μm. **(C)** Quantification of the representative image in **(B)** displayed as proportional location of each c-Fos^*+*^ neuron from the medial edge of MNTB (see the MNTB border and proportional location in Figure 1B). Each point represents one cell. Activated MNTB neurons in *BDNF^+/–^* are more laterally located (median: 24.29% in *BDNF^+/^* and 61.12% in WT; *p* < 0.0001, Mann-Whitney U test). **(D)** Distribution analysis of c-Fos^+^ neurons displayed as the proportional distance from the medial edge of MNTB where 0% is the medial edge of the MNTB and 100% is the most lateral edge of the MNTB. Activated MNTB neurons in *BDNF^+/–^* are more laterally located (*p* < 0.0001, Mann-Whitney U test) and widespread compared to WT (*p* < 0.0001, Kolmogorov-Smirnov test). **** indicates *p* < 0.0001. **(E)** Quantification of c-Fos^+^ neurons divided by total MNTB neurons between genotypes. *BDNF^+/–^* mice (*n* = 5) have larger proportion of c-Fos^+^ MNTB neurons compared to WT (*n* = 3) mice (*p* = 0.018, Wel.ch’s *t*-test). * indicates *p* < 0.05.

Both sexes of wild-type (WT) mice and *BDNF^+/–^* mice with a C57BL/6J background were used under the guidelines approved by the UT Health San Antonio Institutional Animal Care and Use Committee. BDNF heterozygous mice (B6.129S4-*Bdnf^tm1Jae^*/J) were obtained from Jackson Labs in heterozygous breeding pairs. All experiments were done between postnatal days 9 and 11 (P9-P11; Figure 3) and P18-P23 (Figures 1, 2, 4, 5) during the animals’ light cycle. Animals were housed in a 12-h light/dark cycle.

### Sound Stimulation

Mice were exposed to random noise centered at 16 kHz frequency at 80 dB during their light cycle for 3 h per day from P13 to P19 (Figure 4). Sixteen kHz pure tone exposure at 80 dB occurred during the light cycle for 90 min preceded by 60 min of silence in the sound attenuation chamber (Figure 5; Med Associates, Albans, VT) and the stimuli were generated by Tucker Davis Technologies equipment and software.

### Slice Preparation

Mice were anesthetized with isoflurane then rapidly decapitated. The brains were then quickly removed and immersed in ice-cold low-calcium artificial cerebrospinal fluid (aCSF) containing (in mM): 125 NaCl, 2.5 KCl, 3 MgCl_2_, 0.1 CaCl_2_, 25 glucose, 25 NaHCO_3_, 1.25 NaH_2_PO_4_, pH 7.4 bubbled with carbogen (95% O_2_, 5% CO_2_; osmolarity 310–320 mOsm. Transverse 200 μm-thick brainstem slices containing the MNTB were collected using a Vibratome (VT1200S, Leica, Germany). Slices were then prepared further for either electrophysiology or immunohistochemistry experiments.

### Immunohistochemistry

Brain slices were fixed with 4% paraformaldehyde (PFA) for 10 min then washed with PBS three times. Free-floating slices were blocked in 4% goat serum and 0.3% (w/v) Triton X-100, 0.1% Tween 20 in PBS for 1 h and then were incubated with primary antibody overnight at 4°C. Primary antibodies used: Anti-Ankyrin G (Mouse IgG1, NeuroMab, 1:200), Anti-MAP2 (Rabbit or Mouse IgG1, Millipore, 1:500), Anti-β4-spectrin (Rabbit; Bhat lab, UTHSCSA, 1:500), Anti-c-Fos (Rabbit, Synaptic Systems, 1:500). Slices were washed with PBS three times then incubated with corresponding secondary antibodies for 2 h at room temperature. Secondary antibodies (Invitrogen): Alexa Fluor 555 goat anti-GP, Alexa Fluor 488 goat anti-rabbit, and Alexa Fluor 647 goat anti-mouse IgG1 all at 1:500 dilution. Analysis of Z-stack confocal images was performed in Fiji. Medial neurons are in the most medial 30% of the MNTB, and lateral neurons are located in the most lateral 30% of the MNTB. Identification of neuronal AIS utilized either AnkG or β4-spectrin on MAP2^+^ cells. A segmented line tool in Fiji was used to measure length and distance from the soma. AIS length was measured from the proximal end of AnkG^+^ (or β4-spectrin^+^) signal to the most distal end, and AIS location was measured from the proximal end of AnkG^+^ (or β4-spectrin^+^) signal to edge of cell soma. For c-Fos analysis, the double staining against MAP2 and c-Fos were performed from WT and *BDNF^+/–^* mice. MAP2- and c-Fos-positive cells (c-Fos^+^ neurons) were counted using the cell counter plugin of Fiji software. Only the cells with c-Fos^+^ nuclei were counted and the constant threshold level of fluorescence intensity was used in each slice. The percentage of c-Fos^+^ cells was calculated by dividing the number of c-Fos^+^ MAP2^+^ MNTB neurons by the total number of MAP2^+^ MNTB neurons in each slice. Details were described in Kim et al. (2019).

### Electrophysiology

After vibratome sectioning, slices were incubated in a chamber containing normal aCSF bubbled with carbogen at 35°C for 30 min and then kept at room temperature. The normal aCSF was the same as the low-calcium aCSF, except 3 mM MgCl_2_ and 0.1 mM CaCl_2_ were increased to 1 mM MgCl_2_ and 2 mM CaCl_2_. Whole-cell patch-clamp recording was carried out on postsynaptic principal neurons in the MNTB at room temperature (~24°C). Action potentials (APs) were recorded in normal aCSF using the current-clamp mode of the EPC-10 (HEKA Electronik, Lambrecht/Pfalz, Germany). The pipettes were filled with an internal solution containing (in mM) 125 K-gluconate, 20 KCl, 5 Na_2_-phosphocreatine, 10 HEPES, 4 Mg-ATP, 0.2 EGTA, and 0.3 GTP, pH adjusted to 7.3 with KOH. The holding potential was −65 mV in the voltage-clamp mode. Current-clamp protocols were 200 ms in duration with current steps from −100 to 250 pA (50 pA increments). Patch electrodes had resistances of 4–5 MΩ. Series resistance was <15 MΩ without compensation. The threshold of AP was determined by the point where dV/dt exceeds 10 V/s and the amplitude of AP from the threshold to the AP peak in the plot of dV/dt and voltage, which were taken from the first AP evoked in depolarizing current injection protocol. Data were analyzed and displayed with Igor Pro (Wavemetrics, Lake Oswego, OR, United States).

### Western Blot

Whole tissue lysate of WT and *BDNF^+/–^* brainstems (at P21) were extracted with RIPA buffer. Equal protein amounts were loaded onto a 4–15% Tris/Glycine precast gel (Bio-Rad) and transferred to a nitrocellulose membrane. Membranes were blotted with anti-BDNF (Rb, 1:200, Abcam) and anti-β-actin (Mouse, 1:1,000; Abcam), which was used for normalization. Fluorescent secondary antibodies used: Mouse-800CW (1:7,500, LI-COR) and Rabbit-680RD (1:7,500, LI-COR). Membranes were imaged on Odyssey CLx (LI-COR), and images analyzed using Fiji software.

### Quantitative Polymerase Chain Reaction (qPCR)

RNA isolation and reverse transcription reaction were performed as previously described in Kim et al. (2019). RNA extraction was done from p9 whole brain tissue of WT and *BDNF^+/–^* mice using RNAqueous kit with no alterations to procedure (AM1931, Thermofisher). qPCR was executed using 7900HT Fast Real-Time PCR system (Applied Biosystems), data were analyzed using SDS v2.4 (Applied Biosystems). *GAPDH* was used as a reference housekeeping gene. Delta (Δ) CT (Mean_Gene_- Mean_GAPDH_) was utilized to calculate ΔΔCT (ΔCT_Pos_-ΔCT_Neg_), and data was normalized to WT (100%). Mouse primers used: BDNF Forward: 5’-TCGTTCCTTTCGAGTTAGCC, BDNF Reverse: 5’-TTGGTAAACGGCACAAAAC, GAPDH Forward: 5’-AGTATGACTCCACTCACGGCAA, and GAPDH Reverse: 5’-TCTCGCTCCTGGAAGATGGT.

### Statistics

All statistical analyses were performed in GraphPad Prism version 9.2.0 for Windows (GraphPad Software, San Diego, CA, United States). The normality of datasets was analyzed using the Kolmogorov-Smirnov test. Parametric or non-parametric tests were carried out accordingly. To compare the two groups, an unpaired *t*-test with Welch’s correction (parametric) or Mann-Whitney U test (non-parametric) was carried out. To compare three or more groups, one-way ANOVA with Turkey’s multiple comparison test was used. Values in results are represented as mean ± SD. Error bars in figures are ± SD. In Figure 5, Kolmogorov-Smirnov tests are used to evaluate the distribution of c-Fos^+^ cells. Figure 5 contains a box and whisker plot with the median as the center to display neuron distribution within the MNTB.

## Results

### Global Reduction of BDNF Impairs Structural Differentiation of the AIS Along the Tonotopic Axis in the MNTB

The AIS of MNTB neurons is differentiated by structure and function along the tonotopic axis during postnatal development (Kim et al., 2019). To determine whether BDNF mediates the structural refinement of the AIS along the tonotopic axis, we examined the effects of globally reduced BDNF on AIS length and location along the medial-lateral axis in the MNTB using *BDNF*^+/−^ mice. Analysis of *BDNF* mRNA and protein using qPCR and Western Blot respectively showed a significant reduction of *BDNF* mRNA expression and BDNF protein level in *BDNF*^+/−^ mice compared to WT (mRNA: ~75% reduction in *BDNF^+/–^*, *p* = 0.026, Welch’s *t*-test, *n* = 2 and 3; protein: ~22% reduction in *BDNF^+/–^*, *p* = 0.014, Welch’s *t*-test, *n* = 3/group; Figure 1A). In WT and *BDNF*^+/–^ mice (at P20 ± 2 days), we quantified the AIS length and location, measured by the distance from principal neuron soma, as described in Kim et al. (2019). The MNTB was proportionally defined by the percent of the total distance from medial to lateral edges of the MNTB, where medial neurons are within 30% of the medial MNTB border and lateral neurons are within 30% of the lateral MNTB border (Figure 1B). Using MAP2 and AnkG immunostaining, we examined the effect of reduced BDNF on AIS structural properties of MNTB neurons along the tonotopic axis in *BDNF*^+/–^ mice (Figure 1C). In WT mice, the AIS length and location of medial MNTB neurons were significantly different from lateral MNTB neurons. In WT mice, the AIS length was 15.05 ± 2.35 μm (*n* = 59 cells) for medial neurons and 17.43 ± 3.09 μm (*n* = 48 cells) for lateral neurons (*p* < 0.0001; Welch’s 2-tailed *t*-test). The AIS distance from the soma was 10.03 ± 4.10 μm (medial) and 5.51 ± 1.40 μm (lateral, *p* < 0.0001, Welch’s 2-tailed *t*-test; *n* = 27 and 28 cells respectively; Figure 1D). Consistent with our previous work (Kim et al., 2019), AIS length was significantly shorter and more distal from the soma in medial MNTB neurons compared to lateral MNTB neurons. In *BDNF*^+/−^ mice, MNTB neurons did not show this structural differentiation in the AIS length or distance along the tonotopic axis. AIS length was 15.64 ± 3.19 μm in medial neurons and 15.21 ± 2.92 μm in lateral neurons (*p* = 0.43, Student’s *t*-test, *n* = 66 and 64 cells respectively). AIS distance from soma was 6.53 ± 3.29 μm in medial neurons and 6.68 ± 2.99 μm in lateral neurons (*p* = 0.78, Student’s *t*-test, *n* = 56 and 68 cells respectively; Figure 1E). Reduced global BDNF disrupts the tonotopic refinement of AIS properties in the MNTB, indicating that BDNF is associated with the structural development of MNTB neurons. In addition to AIS structural properties, the soma size of MNTB neurons is dependent on location; lateral MNTB neurons are larger than medial neurons (Weatherstone et al., 2017). In the current study, membrane capacitance measurements using whole-cell patch-clamp recordings showed the tonotopic gradient in WT neurons. WT medial neurons had membrane capacitance of 17.02 ± 4.25 pF (*n* = 25), whereas lateral neurons had 20.11 ± 4.65 pF (*n* = 32 neurons, *p* = 0.01, Student’s *t*-test; Table 1). Similarly, *BDNF*^+/−^ mice maintained the tonotopic differentiation of membrane capacitance: medial neurons had a smaller capacitance (13.72 ± 3.20 pF, *n* = 27) than lateral neurons (17.18 ± 3.16 pF, *n* = 37, *p* < 0.0001, Student’s *t*-test; Table 1). Notably, regardless of neuron location, MNTB neurons were significantly smaller in *BDNF*^+/−^ mice compared to WT. Taken together, the reduction of BDNF disrupts the tonotopic differentiation of AIS length and location in the MNTB. The result indicates that globally reduced levels of BDNF impair the structural development of MNTB principal neurons along the tonotopic axis.

**Table 1:** Summary of intrinsic properties of MNTB neurons.

Values represented as Mean ± SD	WT Medial	WT Lateral	BDNF^+/–^ Medial	BDNF^+/–^ Lateral
Rheobase (pA)	112 ± 36.66^A^ *n* = 19	108.4 ± 32.83^A^ *n* = 15	92.86 ± 37.07^A^ *n* = 28	93.42 ± 37.07^A^ *n* = 38
Input Resistance (MΩ)	338 ± 128.7^A^ *n* = 27	301 ± 84.74^A^ *n* = 17	302.6 ± 84.74^A^ *n* = 28	284 ± 123^A^ *n* = 36
Resting Membrane (mV)	−68.58 ± 3.03^A^ *n* = 18	−66.42 ± 1.23^B^ *n* = 15	−66.38 ± 2.12^B^ *n* = 17	−66.40 ± 2.52^B^ *n* = 24
AP Threshold (mV)	−48.98 ± 5.53^A^ *n* = 25	−43 ± 3.06^B^ *n* = 18	−48.87 ± 3.68^AB^ *n* = 26	−47.38 ± 3.68^A^ *n* = 36
AP Amplitude (pA)	73.53 ± 10.22^A^ *n* = 25	72.55 ± 4.69^A^ *n* = 18	75 ± 9.37^A^ *n* = 26	74.4 ± 7.92^A^ *n* = 35
AP Half-width (s)	0.57 ± 0.13^A^ *n* = 18	0.52 ± 0.06^A^ *n* = 15	0.56 ± 0.12^A^ *n* = 16	0.56 ± 0.17^A^ *n* = 20
Capacitance (pF)	17.02 ± 4.25^A^ *n* = 25	20.11 ± 4.56^B^ *n* = 32	13.72 ± 3.21^C^ *n* = 27	17.18 ± 3.16^A^ *n* = 37

*Within rows, means with superscripts containing the same letter are not significantly different. Within the same row, all values with an “A” superscript are not statistically different from each other, and all values with “B” superscript are not statistically different from each other. Within a row, means superscripted with “A” are statistically different from means superscripted with “B”*.

### Tonotopic Organization of MNTB Neuron Intrinsic Properties Is Abolished in *BDNF*^+/–^ Mouse

Next, we investigated how structural alterations of the AIS, caused by BDNF reduction, impact the intrinsic properties and firing pattern of MNTB neurons in *BDNF*^+/−^ mice. APs were recorded from medial and lateral MNTB neurons in response to current injections (from −100 pA to 250 pA, Δ = 50 pA). Phasic plotting the dV/dt of APs from MNTB neurons, evoked by a depolarizing current injection, was analyzed (Figure 2A). In WT mice (at P20), medial neurons had a lower AP threshold and resting potential (more hyperpolarized) compared with lateral neurons, indicating there was a difference in intrinsic properties of MNTB neurons along the tonotopic axis (Threshold, medial: −48.98 ± 5.53 mV and lateral: −41.86 ± 3.06 mV, *p* < 0.0001, Welch’s test; RMP, medial: −68.58 ± 3.03 mV and lateral: −66.42 ± 1.22 mV, *p* = 0.01, Welch’s test). However, there was no difference in AP amplitude, half-width, or input resistance between medial and lateral WT neurons (Figure 2B, Table 1). In *BDNF*^+/−^ mice, there was no difference in AP threshold or resting membrane potential of MNTB neurons along the tonotopic axis (Figures 2C,D). AP threshold was −48.87 ± 3.68 mV (*n* = 26) in medial neurons and −47.38 ± 3.68 mV (*n* = 36) in lateral neurons (*p* = 0.12, Student’s *t*-test). RMP was −66.38 ± 2.12 mV (*n* = 17) in medial neurons and −66.40 ± 2.52 mV (*n* = 24) in lateral neurons (*p* = 0.98, Student’s *t*-test). There was no difference in AP amplitude, half-width, or input resistance between medial and lateral neurons in *BDNF*^+/−^ mice (Figure 2D, Table 1). Thus, global BDNF reduction affects tonotopic differentiation in the AIS and intrinsic properties of MNTB neurons during postnatal development.

Regardless of their location along the tonotopic axis, MNTB neurons from *BDNF*^+/−^ mice showed an increased number of AP spikes evoked by depolarizing current injections (50 pA to 250 pA), compared with WT mice (*p* = 0.0006, ANOVA; Figure 2E). In response to 200 pA current injection (100 ms), the number of spikes was significantly greater in *BDNF*^+/−^ mice (9.4 ± 13.04 APs, *n* = 61 cells) than WT mice (1.76 ± 1.11 APs, *n* = 41 cells, *p* < 0.0001, Welch’s *t*-test), indicating increased excitability in *BDNF*^+/−^ neurons. Rheobase currents were significantly smaller in *BDNF*^+/−^ mice (94.62 ± 36.58 pA) than WT mice (114.6 ± 34 pA, *p* = 0.006, Student’s *t*-test; Figure 2F). The results indicate that reduced BDNF increases the excitability of MNTB neurons regardless of their location.

### At Pre-hearing Age, Reduction of BDNF Does Not Impact Tonotopic Segregation of AIS Structure in the MNTB

BDNF reduction altered structural and physiological development of MNTB neurons that might be dependent on sound-evoked activity after hearing onset at P12. It is possible that BDNF reduction impacts intrinsic properties of MNTB neurons before hearing onset when the auditory processing is dependent on the spontaneous cochlear activity instead of sound-evoked activity. Thus, to test whether a physiological level of BDNF is critical for intrinsic development, which is independent of sound input, we examined the structural properties of the AIS between pre-hearing *BDNF*^+/−^ and WT mice at P9. Pre-hearing WT mice show no tonotopic segregation of AIS properties between medial and lateral neurons (Figures 3A,B), supporting the previous finding that the tonotopic refinement of AIS structures occurs in an activity-dependent manner after hearing onset. However, *BDNF*^+/−^ neurons showed a tonotopic differentiation of AIS length without difference in AIS distance from the soma between medial and lateral neurons. In *BDNF*^+/−^ mice at P9, AIS length was shorter in medial neurons than lateral neurons (17.71 ± 3.39 μm vs. 20.03 ± 4.61 μm, *p* = 0.004, Welch’s two-tailed *t*-test). There was no difference in AIS location along the tonotopic axis (5.29 ± 4.15 μm, *n* = 37 medial vs. 6.48 ± 4.01 μm, *n* = 32 lateral neurons, *p* = 0.23, Student’s *t*-test; Figure 3B).

Next, we examined whether the tonotopic differentiation in the intrinsic properties of MNTB neurons is present in WT and *BDNF*^+/−^ mice at pre-hearing age (Figure 3C). In the whole-cell recording, membrane capacitance did not differ between genotypes in the same position along the tonotopic axis at P9 (Figure 3D). At P9 before hearing onset, the AP threshold was not tonotopically different in WT neurons. However, in *BDNF*^+/−^, lateral neurons showed a higher threshold than medial neurons (Figure 3E). AP threshold was not tonotopically organized in WT mice (*p* = 0.87), but *BDNF*^+/−^ neurons have a tonotopic difference of AP threshold (*p* = 0.01, Welch’s two-tailed *t*-test). Similar to WT, there was no significant difference in AP amplitude between lateral and medial neurons in *BDNF*^+/−^ mice (lateral: 68.97 ± 6.69 mV and medial: 70.53 ± 5.75 mV, *p* = 0.52, Student’s *t*-test). There was no significant difference in AP amplitude between lateral and medial neurons in WT mice (lateral: 68.25 ± 12 mV and medial: 68.98 ± 5.85 mV, *p* = 0.87, Welch’s test). *BDNF*^+/−^ and WT neurons had similar input resistance within the same tonotopic location paralleling the P21 results (Figure 3E). Taken together, MNTB neurons from pre-hearing *BDNF*^+/−^ mice showed tonotopic differences in AIS length and AP threshold. As opposed to unlike in WT mice. However, this tonotopic differentiation did not persist through postnatal development and disappeared after hearing onset (at P21). It suggests that BDNF level may contribute to setting intrinsic properties of MNTB neurons before hearing onset when spontaneous cochlear activity is dominant in the immature auditory system.

### BDNF Is Necessary for Enhanced Tonotopic Refinement Induced by the Sound Augmented Environment

After hearing onset, sound deprivation and enhancement modify structural properties of MNTB neurons during development, and specifically, sound stimulation enhances the tonotopic differences of AIS structure (Kim et al., 2019). We hypothesized that sound stimulation increases BDNF expression which enhances the structural and physiological plasticity of MNTB neurons. Sound stimulation has been shown to increase BDNF transcript and protein levels in the brainstem (Wang et al., 2011; Matt et al., 2018). In WT mice, sound stimulation (80 dB, 16 kHz, 3 h/day) from P13 to P19 increased mature BDNF protein by 25%, but not pro-BDNF, in the auditory brainstem (mature BDNF: *p* = 0.02, Welch’s *t*-test, *n* = 3 per group; pro-BDNF: *p* = 0.40, Welch’s *t*-test, *n* = 4/group; Figure 4A). We examined whether increased endogenous BDNF by sound stimulation rescues the lack of tonotopic differentiation of AIS structure in *BDNF*^+/−^ mice. In *BDNF*^+/−^ mice, which were exposed to the additional sound stimulation (subsequently named *BDNF*^+/−^ + Sound mice), AIS distance from the soma was 6.51 ± 2.21 μm in medial and 6.8 ± 2.20 μm in lateral neurons (*n* = 36 and 34 cells respectively, *p* = 0.58, Student’s *t*-test; Figures 4B,C). AIS length of medial neurons was 15.72 ± 2.24 μm and the length of lateral neurons was 15.58 ± 2.15 μm. AIS length did not differ along the tonotopic axis in *BDNF*^+/−^ + Sound mice (*n* = 35 and 33 cells respectively, *p* = 0.79, Mann-Whitney U test; Figure 4C). The tonotopic difference in soma size was not observed in *BDNF*^+/−^ + Sound mice (Figure 4C). The result demonstrated that augmented sound inputs could not rescue the alterations in AIS structural properties of MNTB neurons caused by global BDNF reduction in *BDNF*^+/−^ mice.

Next, we examined the physiological properties of MNTB neurons in *BDNF*^+/−^ mice following sound stimulation. Phasic plotting the dV/dt of APs from MNTB neurons demonstrated no difference in AP threshold or amplitude between medial and lateral neurons in *BDNF*^+/−^ + Sound (Figures 4D,E). Increased sound input did not induce tonotopic differentiation of neuronal properties in *BDNF*^+/−^ mice. Although exposed to sound stimulation, lack of tonotopic differentiation of AP threshold was maintained in *BDNF*^+/−^ + Sound mice (medial: −45.37 ± 5.75 mV and lateral: −43.98 ± 3.62 mV, *p* = 0.47, Student’s *t*-test; Figure 4E). No significant tonotopic difference was found in AP amplitude or input resistance in *BDNF*^+/−^ + Sound animals (*p* = 0.67, Student’s *t*-test; Figure 4E) like *BDNF*^+/−^ animals. *BDNF*^+/−^ mice were unable to establish MNTB tonotopic gradients of intrinsic or structural properties even when exposed to an augmented sound input during the critical period of development. Mice with BDNF reduction were unable to properly respond to changes in sound-evoked activity, indicating that BDNF is a key molecule for activity-dependent structural and physiological plasticity of auditory brainstem neurons.

### *BDNF^+/–^* Mice Lack Frequency-Responsiveness of Neurons and Show an Impaired Tonotopy in the MNTB

Tonotopy describes a topographic organization of frequency-responsiveness of neurons within each auditory nucleus. To examine if BDNF reduction and associated structural and physiological alterations affect the tonotopy of MNTB neurons, we assessed neuronal activity of MNTB neurons in response to a high frequency tone sound (16 kHz, 90 min, 80 dB) using *c-fos*, an early response gene, immunostaining (Karmakar et al., 2017; Kim et al., 2019; Figure 5A). The expression of c-Fos in the MNTB in response to 16 kHz tone was different between WT and *BDNF^+/–^* mice. In WT mice, c-Fos positive neurons (c-Fos^+^ and MAP2^+^) were mostly located in the medial MNTB, forming a clear band of 16 kHz sound-sensitive neurons, in concordance with high frequency responding neurons residing in the medial portion of the MNTB (Kandler et al., 2009). However, in *BDNF^+/–^* mice, c-Fos^+^ cells were widely spread out across the MNTB without a distinct band-like expression (Figures 5B,C). The distribution analysis for c-Fos^+^ neurons along the tonotopic axis showed the expression of c-Fos in response to 16 kHz sound was specifically concentrated on the medial MNTB from WT mice, but not in *BDNF^+/–^* mice: 0% indicating most medial MNTB position and 100% indicating most lateral MNTB position (WT: 32.36% and *BDNF^+/–^*: 50.21%; *p* < 0.0001, Mann-Whitney U test; Figure 5D). The MNTB from *BDNF^+/–^* mice lack frequency-responsiveness of neurons to 16 kHz sound stimulation, indicating an impaired tonotopy of the MNTB within the auditory brainstem.

To examine neuronal activation in response to 16 kHz sound, we quantified the percentage of c-Fos^+^ neurons (c-Fos^+^ and MAP2^+^ cell #/MAP2^+^ cell # *100) within the MNTB between genotypes. In response to 16 kHz tone, the percentage of c-Fos^+^ cells was significantly higher in *BDNF^+/–^* mice compared to WT (*n* = 5 and 3, respectively; *p* = 0.018, Welch’s *t*-test; Figure 5E), indicating that more MNTB neurons were responsive to the 16 kHz tone sound regardless of their location. The result paralleled physiological properties showing an increased excitability of MNTB neurons in *BDNF^+/–^* mice (Figure 2E). Taken together, *BDNF^+/–^* mice had hyperexcitable MNTB neurons and disorganized isofrequency bands based on the spatial organization of neural activity in response to high frequency sound.

## Discussion

The results demonstrated the role of BDNF in the structural and physiological refinement of MNTB neurons along the mediolateral tonotopic axis using a *BDNF*^+/−^ mouse. Our study is the first *ex vivo* investigation of AIS plasticity with a global reduction of BDNF, which allows us to maintain circuit connections within a sensory system dependent on peripheral inputs. Tonotopy, a driving organization principle of the auditory system, relies on not only sound input but also BDNF signaling to establish proper gradients within the auditory brainstem and ensure precise binaural processing.

### Role of BDNF in the Structural Development of the MNTB

BDNF is an important neurotrophic factor for neural development in an activity-dependent manner, whether that is driven by peripheral inputs or spontaneous activity (Kuczewski et al., 2008; Jiao et al., 2011). We addressed the impact of global BDNF reduction on the structural and physiological refinement of the AIS in a sound input-dependent manner in mature MNTB neurons (at P20) and in a sound input-independent manner in immature neurons in the pre-hearing stage (at P9). Previous works show that tonotopic gradients of K_V_ channel expression and currents require sound input since pre-hearing and congenitally deaf animals do not possess these gradients (von Hehn et al., 2004; Leao et al., 2006; Kim et al., 2019). BDNF is a molecular mediator of neural activity-dependent plasticity shown in LTP of the hippocampus CA1 synapse (Korte et al., 1995; Patterson et al., 1996) and the visual cortex (Akaneya et al., 1997; Huber et al., 1998), as well as structural plasticity of myelin following increased sound input in humans and rats (Bengtsson et al., 2005; de Villers-Sidani et al., 2010). Within the auditory system, BDNF expression is dependent on sound-evoked activity shown with increased BDNF transcript and protein levels in the brainstem following sound stimulation (Wang et al., 2011; Matt et al., 2018). Here we found that at a pre-hearing age when peripheral sound input is not involved in recruiting BDNF, a global reduction of BDNF alters the AIS location of MNTB neurons and abnormally promotes tonotopic differences of AIS length and AP threshold. As opposite to WT mice, tonotopic gradients are present at P9 in *BDNF^+/–^* that are completely abolished by P21. BDNF reduction appears to have disparate effects on tonotopic refinement pre-hearing compared to post-hearing onset, although the underlying mechanism of the disparate effects is not understood. It is known that spontaneous activity driven by supporting cells in the cochlea occurs before hearing onset and allows for intrinsic sound-independent activity throughout the auditory pathway (Sonntag et al., 2009; Babola et al., 2018). Detectable levels of BDNF are present in the brainstem by P6, and spontaneous firing from brainstem neurons drives activity-dependent expression of BDNF before hearing onset (Hafidi, 1999). Thus, the differences were seen in AIS structure between WT and *BDNF*^+/−^ mice before hearing onset could be due to the discrepancy of spontaneous activity between genotypes. BDNF effects on spontaneous activity in pre-hearing age and sound-evoked activity in post-hearing age might be different during development. In the condition with reduced BDNF, spontaneous activity may play a compensatory role in setting intrinsic properties of MNTB neurons, but when switching from spontaneous to sound-evoked activity after hearing onset, this compensatory effect may disappear. It would be worthy to test this hypothesis in future studies.

### BDNF Modulates the Neuronal Activity of MNTB Neurons

The effects of BDNF on neuronal excitability are variable and there are different acute vs. chronic effects. In *BDNF*^+/−^ mice, chronically reduced BDNF levels decreased neuronal activity of pyramidal neurons in the entorhinal cortex (Abidin et al., 2019). We found that regardless of tonotopic location, MNTB neurons from *BDNF*^+/−^ mice showed a hyperexcitability in response to depolarizing current injection and increased neuronal activity in response to high frequency tone exposure compared to WT littermates. Interestingly, an overall increase of neuronal activity was observed, but the tonotopy of the auditory brainstem has been impaired in *BDNF*^+/−^ mice. *In vitro* administration of BDNF increased cell excitability in cultured hippocampal and sensory neurons (Zhang et al., 2008; Guo et al., 2017), but bath perfusion of BDNF reduced excitability of interneurons in the dentate gyrus (Holm et al., 2009; Nieto-Gonzalez and Jensen, 2013). The variable response to BDNF administration might be due to varied ion channel expression, which is influenced by BDNF signaling, across different brain regions.

### Physiological Relevance

How does the disruption of the tonotopic arrangement of neuronal properties impact auditory function? Several genetically modified mice lacking tonotopic gradients of neuronal structure display auditory function abnormalities. CXCR1 mutant mice, which have disrupted microglia-neuron communication, lacked the soma size tonotopic gradient observed in WT mice and had longer peak latencies of the auditory brainstem responses (ABRs) with the normal threshold of ABRs (Milinkeviciute et al., 2021). Ephrin-A3 mutants, which lack a key axon guidance signaling factor, have degraded frequency-responsiveness tonotopy within the cochlear nucleus. These mice have deficits in signal conduction and frequency discrimination with a normal hearing threshold (Hoshino et al., 2021). Outside of genetic modifications, aging disrupts precise tonotopic organization from the cochlear nucleus to the auditory cortex as hearing function degrades (Caspary et al., 2005; de Villers-Sidani et al., 2010). It appears that sound input is required to establish and maintain tonotopy, which is required to maintain proper auditory processing. Thus, it is worthy to further examine whether *BDNF*^+/−^ mice lacking tonotopy of MNTB neurons along the mediolateral axis have auditory processing deficits.

## Data Availability Statement

The raw data supporting the conclusions of this article will be made available by the authors, without undue reservation.

## Ethics Statement

The animal study was reviewed and approved by the UT Health San Antonio Institutional Animal Care and Use Committee.

## Author Contributions

MW and JK contributed to the conception and design of the study. MW collected and analyzed the data. All authors contributed to the article and approved the submitted version.

## Conflict of Interest

The authors declare that the research was conducted in the absence of any commercial or financial relationships that could be construed as a potential conflict of interest.

## Publisher’s Note

All claims expressed in this article are solely those of the authors and do not necessarily represent those of their affiliated organizations, or those of the publisher, the editors and the reviewers. Any product that may be evaluated in this article, or claim that may be made by its manufacturer, is not guaranteed or endorsed by the publisher.
